# *Moringa oleifera* Leaf Extract Protects C2C12 Myotubes against H_2_O_2_-Induced Oxidative Stress

**DOI:** 10.3390/antiox11081435

**Published:** 2022-07-24

**Authors:** Roberta Ceci, Mariateresa Maldini, Mark E. Olson, Domenico Crognale, Katy Horner, Ivan Dimauro, Stefania Sabatini, Guglielmo Duranti

**Affiliations:** 1 Laboratory of Biochemistry and Molecular Biology—Department of Movement, Human and Health Sciences, Università degli Studi di Roma “Foro Italico”, Piazza Lauro De Bosis 6, 00135 Roma, Italy; roberta.ceci@uniroma4.it (R.C.); stefania.sabatini@uniroma4.it (S.S.); 2 SCIEX Italia S.r.l., Via Montenapoleone, 8, 20121 Milano, Italy; mariateresa.maldini@sciex.com; 3 Instituto de Biología, Universidad Nacional Autónoma de México, Tercer Circuito de CU s/n, Ciudad de México 04510, Mexico; molson@ib.unam.mx; 4 Institute for Sport & Health, School of Public Health, Physiotherapy and Sports Science, University College Dublin, D04 V1W8 Dublin, Ireland; domenico.crognale@ucd.ie (D.C.); katy.horner@ucd.ie (K.H.); 5 Laboratory of Biology and Human Genetics—Department of Movement, Human and Health Sciences, Università degli Studi di Roma “Foro Italico”, Piazza Lauro De Bosis 6, 00135 Roma, Italy; ivan.dimauro@uniroma4.it

**Keywords:** *Moringa oleifera* leaf extract (MOLE), C2C12 skeletal muscle cells, redox status, enzymatic antioxidant system, oxidative stress

## Abstract

The imbalance between reactive oxygen species (ROS) production and antioxidant defense systems leads to macromolecule and tissue damage as a result of cellular oxidative stress. This phenomenon is considered a key factor in fatigue and muscle damage following chronic or high-intensity physical exercise. In the present study, the antioxidant effect of *Moringa oleifera* leaf extract (MOLE) was evaluated in C2C12 myotubes exposed to an elevated hydrogen peroxide (H_2_O_2_) insult. The capacity of the extract to influence the myotube redox status was evaluated through an analysis of the total antioxidant capacity (TAC), glutathione homeostasis (GSH and GSSG), total free thiols (TFT), and thioredoxin (Trx) activity, as well as the enzyme activities of superoxide dismutase (SOD), catalase (CAT), and glutathione peroxidase (GPx) and transferase (GST). Moreover, the ability of MOLE to mitigate the stress-induced peroxidation of lipids and oxidative damage (TBARS and protein carbonyls) was also evaluated. Our data demonstrate that MOLE pre-treatment mitigates the highly stressful effects of H_2_O_2_ in myotubes (1 mM) by restoring the redox status (TFT, Trx, and GSH/GSSG ratio) and increasing the antioxidant enzymatic system (CAT, SOD, GPx, GST), thereby significantly reducing the TBARs and PrCAR levels. Our study provides evidence that MOLE supplementation has antioxidant potential, allowing myotubes better able to cope with an oxidative insult and, therefore, could represent a useful nutritional strategy for the preservation of muscle well-being.

## 1. Introduction

Skeletal muscles represent 30–40% of the total body mass and have a key role in the well-being of the entire organism. They are involved in movement, heat production, breathing, and have a primary role in maintaining glycemic levels and an efficient body energy balance. Skeletal muscles are very variable in ATP request, varying from a resting, low-energy consuming condition to a very high-energy request following intense muscle contraction [1]. To fulfill energy demand, more oxygen is released from hemoglobin, and in mitochondria, oxidative phosphorylation increases their working capacity, producing the necessary ATP [2]. At the same time, mitochondria are also the main reactive oxygen species (ROS) generators through the leak of electrons in complexes I and III of the electron transport chain [3].

Among ROS, hydrogen peroxide (H_2_O_2_) is a Janus-faced molecule. It exerts an opposite role on skeletal muscle function depending on its concentration [4,5,6,7]. Levels from low to moderate act as signals for cell adaptation and are necessary for muscle growth and repair [4,5,6,7]. On the contrary, high levels of H_2_O_2_ with the consequent formation of oxidized macromolecules may contribute to the loss of myoblast function, increase cell death, and worsen muscle-repair mechanisms [8,9,10]. Muscle contractions during physical exercise, especially intense or unaccustomed, are usually accompanied by the high production of ROS that ultimately leads to oxidative stress, which potentially results in myofiber damage evidenced by increased biomarkers of oxidation in both skeletal muscle and blood [11,12]. Moreover, oxidative stress is one of the key factors for the development of fatigue, a phenomenon that many sports practitioners experience and that leads to a deterioration of exercise performance. In addition, oxidative stress has been reported as being involved in such diverse phenomena as aging, diabetes mellitus, cancers, and Alzheimer’s disease [13,14]. 

In physiological conditions, ROS are maintained at a low level by the action of several types of antioxidants. Among them, an important role is played by natural dietary antioxidants (e.g., vitamins, polyphenols, flavonoids), endogenous antioxidant enzymes (e.g., superoxide dismutase (SOD), catalase (CAT), glutathione peroxidase (GPx), glutathione transferase (GST)), and endogenous antioxidant molecules (mainly by the thiol system, by glutathione (GSH) and thioredoxin (Trx)) [15,16,17,18,19,20]. 

The disruption of the homeostasis of cellular antioxidant systems is the most central feature of oxidative stress occurrence. In the effort to prevent and/or contain such harmful imbalances, researchers have investigated nutritional strategies to improve physical capabilities, such as a reduction in fatigue and increased exercise endurance [21,22,23,24]. 

During recent decades, the natural products of *Moringa oleifera* Lam. (Family Moringaceae; Order *Brassicaceae*) have been extensively investigated in various biological systems [25,26,27,28,29]. 

It has been shown that *Moringa oleifera* has antioxidant features that are due to the many bioactive compounds present in different parts of the plant. Specifically, tannins, saponins, flavonoids, and terpenoids are very well represented, especially in the leaves. These molecules demonstrate beneficial features acting as antioxidants, and/or antimicrobial, and/or anti-carcinogenic agents [30,31]. Moreover, these molecules have proven themselves to be effective in treating several chronic pre-pathological conditions. In fact, hypercholesterolemia, insulin resistance, and inflammation, whose onset is based on the increase in reactive oxygen species, have been shown to be reduced by the effect of flavonoids and other glycosides [32,33,34,35]. Moreover, phenolic acids (e.g., chlorogenic acid and ferulic acid) are also present at moderate concentrations in extracts of *Moringa oleifera* leaves. They are known to act as primary antioxidants, for example, by inactivating lipid free radicals or by acting in the prevention of the decomposition of hydroperoxides into free radicals [36,37,38]. 

Recently we demonstrated that *Moringa oleifera* leaf extract (MOLE) improved oxidative capacity in C2C12 myotubes by the activation of the SIRT1-PPARα pathway leading the cells to an increased reliance on lipid metabolism [36]. Moreover, MOLE has a beneficial effect on the antioxidant system of skeletal muscle cells through the induction of the Nrf2-HO-1 pathway [29]. 

Based on these findings, we have hypothesized that MOLE, having the ability to switch on the pathways that upregulate the antioxidant defense system, could efficiently protect skeletal muscle cells subjected to a pro-oxidant environment. 

In the present study, the goal is to provide scientific evidence regarding the effect of *Moringa oleifera* leaf extract on restoring redox balance after a strong oxidative insult that mimicks the distress condition that happens in high, intense muscle contractions. To this end, differentiated C2C12 skeletal muscle cells were treated with MOLE for 24 h and then exposed to 1 mM H_2_O_2_ for 1 h and analyzed for: (a) cell viability and the total antioxidant capacity (TAC), an assay that measures lipo- and hydro-philic antioxidants; (b) GSH homeostasis, the level of intracellular free thiols and the activity of thioredoxin, as markers of redox status; (c) antioxidant enzymatic defense network: superoxide dismutase (SOD), catalase (CAT), glutathione peroxidase (GPx), and glutathione transferase (GST) activities; and (d) protein carbonyls (PrCar) and lipid peroxidation (TBARS) as markers of oxidative damage.

## 2. Materials and Methods

All chemical reagents, unless otherwise specified, were purchased from Sigma-Aldrich Chemical (St. Louis, MO, USA).

### 2.1. MOLE: Methanolic Extract of Moringa oleifera Leaves

One gram of *Moringa oleifera* leaf powder (PureBodhi Nutraceuticals Ltd., London, UK) was dissolved in 10 mL of methanol (100%) and then sonicated (Vibra-Cell CV 18 SONICS VX 11, Sonics & Materials, Newtown, CT, USA) twice for 10 min at +4 °C. The obtained extract was then centrifuged (2000× *g* for 10 min at +4 °C) and then collected and stored at −20 °C (stock solution).

### 2.2. MOLE Qualitative Profiling

Qualitative profiling of the tested MOLE extract was obtained by ultra-high performance liquid chromatography-quadrupole time-of-flight mass spectrometry (UHPLC/Q-TOF-MS, SCIEX X500B, AB SCIEX GmbH, Landwehrstraße 54, Darmstadt, Germany) technique with a high-resolution SCIEX X500B QTOF electrospray ion source operated in negative ion mode as previously described [39]. The digital fingerprint of the sample was characterized with SWATH^®^ analysis.

The data obtained were processed using SCIEXOS Software 2.1 (AB SCIEXGmbH, Landwehrstraße 54, Darmstadt, Germany), and the SCIEX Natural Products 2.1 Library (AB SCIEX GmbH, Landwehrstraße 54, Darmstadt, Germany) was used to search for database compound spectra.

### 2.3. Cell Cultures

C2C12 myoblasts (ATCC, Manassas, VA, USA) were cultured, as previously described [29]. Preconfluent cells (85% confluency) were induced to differentiate by lowering the FBS to 2% in a culture medium. Cell differentiation was monitored by microscopy and assessed by myogenin and MHC expression by Western blot analysis [40]. 

C2C12 myotubes were treated with H_2_O_2_ dose-dependently (0.1–1 mM) for 1 h to verify cytotoxicity. The methyl-thiazolyl-diphenyl-tetrazolium bromide (MTT) assay was performed to test cell viability [41]. Then, 1 mM H_2_O_2_ was chosen for further experiments. Then, the cells were treated with working solutions with 1/1000 or 1/100 MOLE solutions or vehicle-only (methanol) in a culture media for 24 h. In these working solutions, the methanol concentration (0.1%, *v*/*v*) does not have any specific effect on myotubes. 

Subsequently, hydrogen peroxide (1 mM) was added to samples pre-treated with vehicle or MOLE for a further hour, MTT assays were performed, and the samples were prepared for biochemical analysis.

The cells were trypsinized, collected, and centrifuged at 1200× *g* for 10 min at room temperature. Gentle lysis was then performed, and the lysate obtained was then used for biochemical analysis or tested for protein content using the Bradford method (Sigma-Aldrich, St. Louis, MO, USA).

### 2.4. TAC: Trolox^®^ Equivalents Antioxidant Capacity

The total antioxidant capacity (TAC) was performed spectrophotometrically by the Trolox^®^ equivalents antioxidant capacity assay, as previously described [42]. This assay evaluates the ability of cell lysates to prevent ABTS^+^ radical formation in ABTS-metMyo-PBS buffer after the addition of H_2_O_2_ (450 μM) compared to the vitamin E analogue Trolox^®^ standards. 

The variation in absorbance detected at 734 nm was compared to those obtained using Trolox^®^ standards (0.125–2.0 mM) and expressed as micromoles/mg of protein tested. 

To check the efficiency of the extraction method, 10 μl of different MOLE stock solution dilutions (0.015, 0.075, 0.15, and 1.5 mg/mL of dried powder corresponding to 1/1000–1/500–1/100, and 1/10 working solution, respectively) were tested and the antioxidant capacity obtained were comparable with those already reported [29,39] (data not shown).

### 2.5. GSH, GSSG and GSH/GSSG Evaluation

Myotubes reduced (GSH), oxidized (GSSG) glutathione contents, and the GSH/GSSG ratio were quantified by a DTNB–glutathione reductase recycling assay, as previously described [43]. 

GSSG was selectively measured in samples in which reduced glutathione was masked using a 2-vinylpyridine (2%) pretreatment. The variation in TNB formation absorbance was followed at 412 nm and compared to those obtained by using glutathione standards, and the results were normalized for protein content.

### 2.6. Total Free Thiol Levels and Thioredoxin Activity Analysis

The cellular *total free thiol* concentration was quantified by the Ellman assay, as previously described [44], by following the TNB anion formation upon the reaction of thiols with DTNB. The mean intrinsic absorbance was subtracted from the mean absorbance of TNB release. The molar concentration of the thiols was calculated from the molar absorbance of the TNB anion and expressed as micromole –SH/g cell lysate amounts [44].

*Thioredoxin activity* was measured using the insulin disulfide reduction assay according to Holmgren and Björnstedt [45]. The total cellular protein lysate was incubated with reaction buffer (50 mM HEPES pH 7.6, 1 mM EDTA, 1 mg/mL BSA, 2 mM DTT) at 37 °C for 15 min and then incubated with thioredoxin reductase (American Diagnostica Inc., Greenwich, CT, USA) in a reaction buffer (20 mM HEPES pH 7.6, 1 mM EDTA, 200 μM NADPH and 0.3 mM insulin) at 37 °C for 20 min. After that, the stop mix buffer (6 M guanidine HCl, 1 mM DTNB in 0.2 M Tris-HCl pH 8.0) was added, and the absorption at 412 nm was measured. The Trx activity level was compared with standards and expressed as micrograms of Trx per milligram of the total cellular proteins tested.

### 2.7. Lipid and Protein Oxidation

*Thiobarbituric acid reactive substances.* The TBARS levels were assayed by spectrophotometric analysis [46]. The methodology measures malondialdehyde (MDA) and other aldehydes produced by lipid peroxidation induced by hydroxyl free radicals. Sample absorbance was determined spectrophotometrically at 535 nm and compared to a standard MDA (1,1,3,3-tetramethoxypropane) solution. The levels of TBARS were expressed in terms of nmol/mg protein [46].

*Protein carbonyls.* The protein carbonyl levels (PrCAR) were determined by measuring the reactivity of carbonyl derivatives with 2,4-dinitrophenylhydrazine (DNPH) according to Colamartino et al. [47]. The protein carbonyl content was calculated from the absorbance measurement of the conjugated DNTB at 380 nm. The millimolar extinction coefficient for DNPH is ε380 = 22.00. The PrCAR content was expressed in terms of nmol/mg protein [47].

### 2.8. Antioxidant Enzymatic Activities

Intracellular *superoxide dismutase*, *catalase*, and *glutathione peroxidase* activities were assayed spectrophotometrically using commercial assay kits (Cayman Chemical Company, Ann Arbor, MI, USA) following the manufacturer’s instructions and the results were expressed as the units/milligrams of protein tested as previously described [44,46,48].

Intracellular *glutathione transferase activity* was assayed with spectrophotometric methods as previously described [49]. Cell extracts (100 μL) were incubated with 1 mM glutathione and 1 mM of 1-chloro-2,4-dinitrobenzene in 1 mL of 0.1 M potassium-phosphate buffer, pH 6.5. S-glutathionyl-2,4-dinitrobenzene formation was monitored at 340 nm (ε340 nm = 9600 M/cm). For each spectrophotometric determination, the spontaneous reaction of glutathione with 1-chloro-2,4-dinitrobenzene was subtracted. The GST activity was expressed in enzymatic units (U) at 37 °C and normalized for protein content.

### 2.9. Statistical Analysis

The distribution of the data was evaluated using the Kolmogorov–Smirnov test. All of the data are expressed as the means ± S.D. of three independent experiments, each performed in triplicate. One-way ANOVA for repeated measures and Bonferroni post-hoc analyses were performed in order to determine significant variation among groups for each variable tested. A value of *p* < 0.05 was accepted as statistically significant. The SPSS program for Windows (Version 17.0; SPSS Inc., Chicago, IL, USA) was used for all statistical analyses. The analysis performed between untreated controls and control vehicles (CTRLm) showed no statistical differences for all variables tested (data not shown).

## 3. Results

### 3.1. MOLE Metabolomic Fingerprint

UHPLC-MS analysis highlights the presence of four main secondary metabolite groups in MOLE extract: glucosinolates, flavonoids, polyphenols, and phenolic acids. A digital record of the sample was acquired by SWATH^®^ analysis with MS/MS spectral information for all of the detectable precursor ions in the defined mass range. SCIEX Natural The Products Library 2.1, a database for potential compound identification (library match score > 75% and mass error +/−2 ppm), was used for compound identification based on the product’s ion spectral information. About 27 secondary metabolites among flavonoids, polyphenols, and phenols were identified with a high library score (78–100%) [39]. 

**Figure 1:**
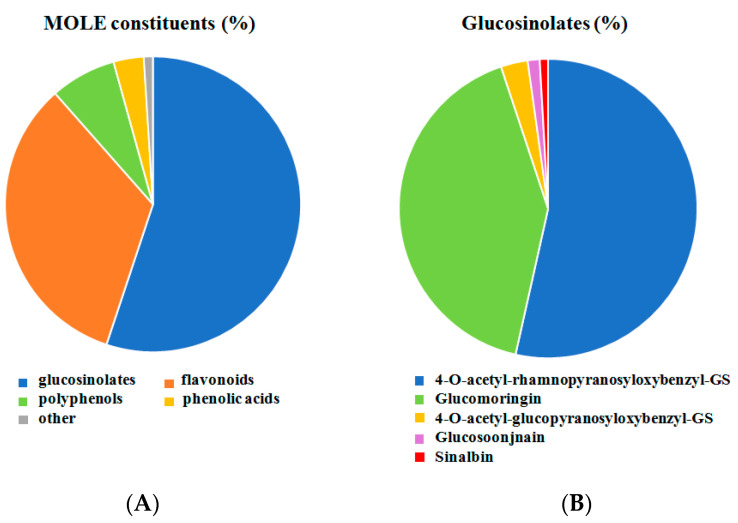
**Relative amounts of constituents of MOLE.** Metabolomic analysis was performed on MOLE working solution. Relative percent of different categories of compound was shown in panel (**A**). SWATH MS/MS spectrum of MOLE extract was performed for glucosinolates analysis. Relative percent were shown in panel (**B**).

Figure 1A shows the relative percentage of metabolite categories. Glucosinolates (55.1%), flavonoids (33.4%), polyphenols (7.2%), and phenolic acids (3.3%) were the most highly represented in the metabolomic fingerprint. 

The most intense peaks were represented by GLs. The lack of reference standards for some GLs and as a consequence of their MS/MS spectral information, a loss of number in the number of identified GLs may occur. Then, a different approach was undertaken for their identification. These molecules are thioglucoside compounds containing a sulfated aldoxime moiety. Moreover, a variable side chain derived from amino acids is present. Glucosinolates have a particular chemical structure producing diagnostic typical fragment ions in the MS/MS spectra. In particular, the GLs sulfated glucose moiety (fragment 259.01 *m/z*) and a sulfate group (fragment 96.96 *m/z*) could be identified in spectra and assigned [50,51].

Glucosoonjnain (tR: 3.8; 1.3%), glucomoringin (tR: 4.2; 41.4%), sinalbin (tR: 4.3; 0.9%), 4-O-acetyl-rhamnopyranosyloxybenzylGS (tR: 6.0, 6.4, 7.7; 53.5%), and 4-O-acetyl-glucopyranosyloxybenzylGS (tR: 5.3, 7.0; 2.9%). were identified from ion chromatograms XIC, MS, and SWATH MS/MS spectrum for identified glucosinolates and expressed as a percentage (Figure 1B).

### 3.2. Cell Viability

Cell viability was assessed by the methylthiazolyldiphenyl-tetrazolium bromide (MTT) assay [52]. 

The cells were treated with a range of H_2_O_2_ (0.1–1 mM, dose-dependence) for 1 h. 

**Figure 2:**
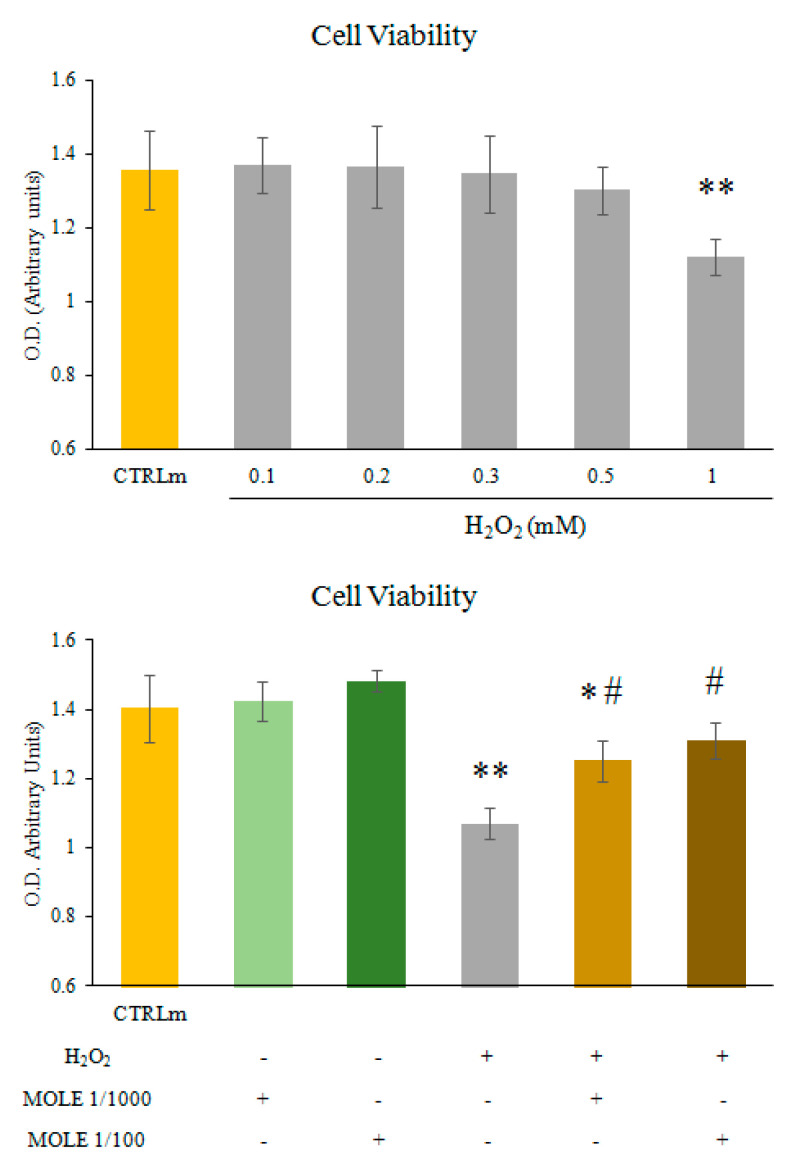
**MTT assay.** C2C12 myotubes were treated with different H_2_O_2_ concentrations (0.1–1 mM) for 1 h (dose-dependence, upper panel). Then the effects of MOLE pre-treatments were assayed. C2C12 myotubes were treated with MOLE stock solution dilutions (1/1000 and 1/100 working solution) or vehicle (methanol) in culture media for 24 h. Then, hydrogen peroxide (1 mM) was added to samples pre-treated with vehicle or MOLE for a further hour (lower panel). Cell viability was assessed by the MTT assay. Data presented are the mean ± S.D. of three experiments, each performed in triplicate. * *p* < 0.05; ** *p* < 0.01 vs. CTRLm; # *p* < 0.05 vs. H_2_O_2_.

We found that H_2_O_2_ 1 mM induced a statistically significant reduction in myotube viability (Figure 2, upper panel). Therefore, 1 mM of H_2_O_2_ was selected for all of the subsequent experiments. To test the protective effect of MOLE, C2C12 myotubes were treated with MOLE stock solution dilutions (1/1000 and 1/100 working solution) or a vehicle (methanol) in culture media for 24 h. 

After incubation, hydrogen peroxide (1 mM) was added to the samples pre-treated with vehicle or MOLE for a further hour, and an MTT assay was performed (Figure 2, lower panel). No statistically significant differences were found between the single MOLE treatments. We found that myotubes pre-treated with MOLE and then exposed to H_2_O_2_ showed markedly greater cell viabilities, of 17% and 22%, compared to single H_2_O_2_ for 1/1000 and 1/100 dilutions of MOLE, respectively (Figure 2, lower panel).

### 3.3. Evaluation of Glutathione Homeostasis and Total Antioxidant Capacity

**Figure 3:**
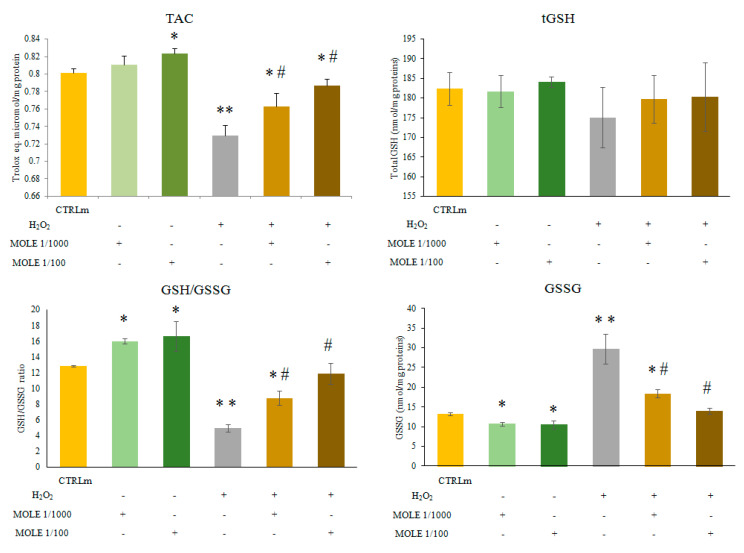
**Total antioxidant capacity (TAC) and glutathione homeostasis analysis.** Measurement of total antioxidant capacity (TAC), total glutathione (tGSH), oxidized glutathione (GSSG), and reduced to oxidized glutathione ratio (GSH/GSSG) was performed in C2C12 myotubes treated with MOLE stock solution dilutions (1/1000 and 1/100 working solution) or vehicle (methanol) in culture media for 24 h and in samples treated for a further hour with hydrogen peroxide (1 mM) with pre-treatment with vehicle or MOLE. Data presented are the mean ± S.D. of three experiments. * *p* < 0.05; ** *p* < 0.01 vs. CTRLm; # *p* < 0.05 vs. H_2_O_2_.

The evaluation of glutathione homeostasis revealed significant differences in the GSH/GSSG ratio, a well-known marker of redox status, between the different treatments. The MOLE treatment markedly decreased the GSSG levels (19% and 20% for MOLE 1/1000 and 1/100, respectively, *p* < 0.01) compared to CTRLm (Figure 3).

The H_2_O_2_ (1 mM, 1 h) treatment increased the GSSG levels (225% *p* < 0.01) compared to CTRLm (Figure 3). The MOLE pre-treatment decreased the H_2_O_2_-induced rise in GSSG levels in a statistically significant manner (38% and 53% for MOLE 1/1000 and 1/100, respectively, *p* < 0.01) compared to H_2_O_2_ (Figure 3). 

No statistically significant differences were found after treatments in the total glutathione (tGSH) levels. Only a slight, non-significant decrease was found after the H_2_O_2_ treatments.

No statistically significant differences were found after treatments in the total glutathione (tGSH) levels. Only a slight and non-significant decrease was found after H_2_O_2_ treatments. 

Because the total glutathione levels were not affected by the treatments, the evaluation of the ratio between the reduced and oxidized glutathione (GSH/GSSG) showed a statistically significant increase after the MOLE treatment (25% and 29% for MOLE 1/1000 and 1/100, respectively, *p* < 0.01) compared to CTRLm (Figure 3).

The 1-h H_2_O_2_ 1 mM treatment induced a decrease in the GSH/GSSG ratio (61% *p* < 0.01) compared to CTRLm (Figure 3). The pre-treatment with MOLE increased the H_2_O_2_-induced fall in GSH/GSSG ratio in a statistically significant manner (177% and 239% for MOLE 1/1000 and 1/100, respectively, *p* < 0.01) compared to H_2_O_2_ (Figure 3). 

The analysis of myotube total antioxidant capacity showed a statistically significant increase after MOLE 1/100 treatment (*p* < 0.05) and a reduction after H_2_O_2_ treatment (*p* < 0.01). The pre-treatment with MOLE was able to partially revert in a statistically significant manner the H_2_O_2_-induced decrease in total antioxidant capacity (*p* < 0.05 for MOLE 1/1000 and *p* < 0.01 for MOLE 1/100; Figure 3).

### 3.4. Total Free Thiols and Thioredoxin Activity Analysis

Free thiol residues play a fundamental role in the homeostasis of the cellular redox state and in the detoxification of reactive oxygen species [53]. We investigated whether MOLE was able to keep these groups in the reduced-activity state. The MOLE treatment did indeed induce an increase in the myotube total free thiol levels (7% and 10% for MOLE 1/1000 and 1/100, respectively, *p* < 0.05) compared to CTRLm (Figure 3). Similarly, 1-h H_2_O_2_ 1 mM treatment induced a decrease in TFT levels (*p* < 0.05) compared to CTRLm (Table 1). Finally, a pre-treatment with MOLE was able to revert the H_2_O_2_-induced decrease in TFT levels in a statistically significant manner (7% and 13% for MOLE 1/1000 and 1/100, respectively, *p* < 0.05) compared to H_2_O_2_ (Table 1). 

The same effects were found in the analysis of the total levels of thioredoxin and its reactive active form. MOLE was able to increase the total Trx (10% and 19% for MOLE 1/1000 and 1/100, respectively, *p* < 0.05) and active Trx levels (27% and 38% for MOLE 1/1000 and 1/100, respectively, *p* < 0.05), and in pre-treated myotubes, MOLE was able to revert the H_2_O_2_-induced decrease in total Trx and active Trx levels in a statistically significant manner (9% and 23% for MOLE 1/1000 and 1/100, respectively, for total Trx, *p* < 0.05; and 69% and 92% for MOLE 1/1000 and 1/100, respectively, for active Trx, *p* < 0.05) compared to H_2_O_2_ (Table 1).

### 3.5. Evaluation of Antioxidant Enzyme Activities

Compared to CTRLm, MOLE was able to increase the activity of all of the antioxidant enzymes in C2C12 myotubes. 

MOLE administration induced a treatment effect (*p* < 0.05) and a dose-dependent increase in SOD (8% and 24% for MOLE 1/1000 and 1/100, respectively) and GST (11% and 17% for MOLE 1/1000 and 1/100, respectively) (*p* < 0.05, Table 2). A treatment effect was found for CAT (13% and 17% increase for MOLE 1/1000 and 1/100, respectively) and GPx (27% and 31% increase for MOLE 1/1000 and 1/100, respectively; *p* < 0.05, Table 2). However, no dose effect was found for these enzymatic activities (Table 2).

The H_2_O_2_ (1 mM, 1 h) treatment induced a decrease in all enzymatic activity levels compared to CTRLm (SOD: 17%, CAT: 14%, GPx: 15%, GST: 15%; *p* < 0.01, Table 2).

MOLE pre-treatment was able to revert the decrease in the activities H_2_O_2_-induced for all enzymes in a statistically significant manner when compared to H_2_O_2_ (21% and 24% increase for MOLE 1/1000 and 1/100, respectively, for SOD; 22% and 28% increase for MOLE 1/1000 and 1/100, respectively, for CAT; 30% and 38% increase for MOLE 1/1000 and 1/100, respectively, for GPx; 23% and 29% increase for MOLE 1/1000 and 1/100, respectively, for GST; *p* < 0.05; Table 2). 

### 3.6. Evaluation of Oxidative Damage Markers

**Figure 4:**
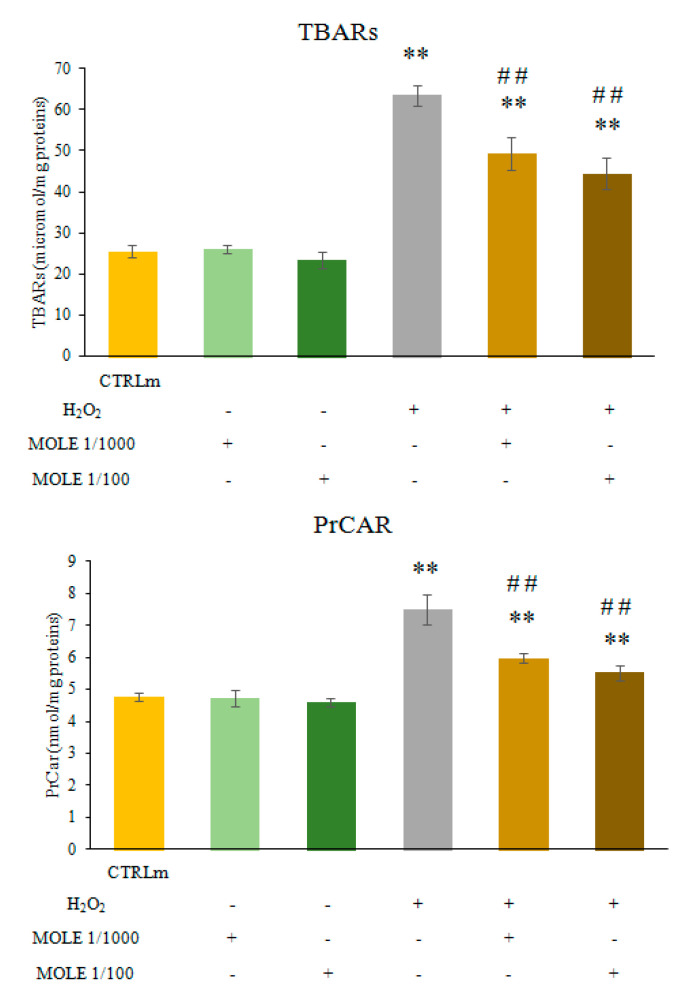
**Thiobarbituric acid reactive substances (TBARs) and protein carbonyls (PrCar) analysis.** Measurement of TBARs and PrCar analysis was performed in C2C12 myotubes treated with MOLE stock solution dilutions (1/1000 and 1/100 working solution) or vehicle (methanol) in culture media for 24 h and in samples treated for a further hour with hydrogen peroxide (1 mM) with pre-treatment with vehicle or MOLE. Data presented are the mean ± S.D. of three experiments. ** *p* < 0.01 vs. CTRLm; ## *p* < 0.01 vs. H_2_O_2_.

As shown in Figure 4, the MOLE treatment did not induce any significant change in PrCAR and TBARs (malondialdehyde (MDA) and other aldehydes) levels if compared to untreated cells. H_2_O_2_ (1 mM, 1 h) exposure induced a statistical increase in protein carbonyls and TBARs (172% and 249%, respectively; *p* < 0.01) compared to CTRLm (Figure 4).

MOLE pre-treatment was able to lower the H_2_O_2_-induced increase in oxidative damage markers in a statistically significant manner (*p* < 0.01) compared to H_2_O_2_ (27% for both MOLE 1/1000 and 1/100 for PrCAR, *p* < 0.01; and 23% and 30% for MOLE 1/1000 and 1/100, respectively, for TBARs, *p* < 0.01; Figure 4). 

## 4. Discussion

In the present study, we found that MOLE efficiently counteracts the oxidative damage induced by a high dose of hydrogen peroxide in C2C12 myotubes. MOLE pretreatment blunts the oxidative insult by stimulating antioxidant enzyme activities and restoring redox status, thereby limiting the damage to lipids and proteins.

Skeletal muscle is a tissue that is often exposed to pro-oxidizing conditions due to its high oxygen consumption rates. [54,55]. In fact, every increase in metabolic activity leads to increased levels of ROS. In physiological amounts, they regulate numerous cellular signaling pathways and modulate the expression of many redox-sensitive genes. ROS production is usually matched by endogenous antioxidant defenses, but if ROS are in excess, oxidative stress occurs, predisposing organisms to pathological conditions [56,57,58]. 

Our previous data show that, in C2C12 myotubes, treatment with MOLE caused the activation of the oxidative metabolism through the SIRT1-PPARα pathway along with the activation of the nuclear factor erythroid 2-related factor (Nrf2) and its target gene heme oxygenase-1 (HO-1), both regulators of cellular resistance to oxidants. MOLE treatment counterbalanced the stimulated oxidative metabolism with an improved glutathione redox homeostasis and increased antioxidant enzymatic activities [29,39]. 

It is well established that vigorous, unaccustomed physical exercise increases muscle energy requests enormously, leading to very high anion superoxide (O2^°-^) production by mitochondria and NADPH oxidase activities. The successive dismutation of O2^°-^ leads to uncontrolled, high intracellular levels of H_2_O_2_, a condition that sportsmen take pains to avoid because it results in contractile dysfunction and fatigue. In this situation, cell damage occurs due to the impairment of the redox state and the functionality of various proteins, including antioxidant enzymes, up to the events that lead to cell death [5,6,7,59,60].

Examining the effect of H_2_O_2_ in vitro at the cellular level, we have shown that the exposure of C2C12 myotubes to H_2_O_2_ 1 mM decreased cell viability, antioxidant capacity, GSH/GSSG ratio (a primary marker of redox status), and the levels of total free thiols and thioredoxin. The disruption of the homeostasis of myotube thiol systems is the most central feature of the oxidative stress occurrence induced by H_2_O_2_ exposure. Moreover, the levels of superoxide dismutase, catalase, glutathione peroxidase, and glutathione transferase activities were also found to be decreased by H_2_O_2_ exposure. The adverse effects of H_2_O_2_ were mirrored by an increase in oxidative damage markers such as PrCAR and TBARs. 

To preserve muscle function and protect myotubes from excessive exposure to ROS, the use of antioxidants is a common strategy, and our results show that *Moringa* offers a useful resource for such strategies. The appropriate use of antioxidants has proved to be beneficial in balancing the ratio between oxidants and antioxidants in most physio-pathological conditions [21,61,62].

*Moringa oleifera* leaf extracts are rich in glucosinolates, polyphenols, flavonoids, and phenolic acids that are known to act as antioxidants, either inactivating lipid free radicals or preventing the decomposition of hydroperoxides into free radicals [36,37,38].

Following these findings, here we examined whether the antioxidant molecules in *Moringa oleifera* leaf extracts are present in sufficient concentrations to counteract the damage provoked by the oxidizing-/oxidative environment in myotubes exposed to 1 mM H_2_O_2_. We have previously observed that MOLE showed, per se, in a cell-free system a dose-dependent total antioxidant capacity indicating that its ability depends on the amount of antioxidant molecules present in the mixture [39]. 

Further, since our preliminary data highlighted the incapacity of MOLE to exert a significant protective effect on C2C12 myotubes when added at the same time point of H_2_O_2_ treatment (data not shown), we adopted a pre-supplementation strategy by which MOLE was added in culture medium 24 h before the acute H_2_O_2_ treatment.

Our data demonstrate that the presence of MOLE restores the redox status as evidenced by blunting the negative effects of H_2_O_2_ on the intracellular GSH/GSSG ratios, the level of free thiols, and the activity of thioredoxin, and furthermore positively modulates antioxidant enzyme activities. These features lead to a decrease in H_2_O_2_-induced oxidative damage with the result of improving myotubes viability. 

The pre-treatment of myotubes with MOLE increased free thiols, thioredoxin activity, and intracellular TAC. The TAC assay allows for the determination of the levels of the total non-enzymatic antioxidant capacity of biological samples in terms of lipid-soluble antioxidants such as tocopherols, carotenes, vitamin A, ubiquinols, and water-soluble antioxidants such as glutathione, as well as ascorbate and proteins with redox-sensitive thiols. In particular, the levels of intracellular free thiols are a fundamental component for maintaining the redox state and the total antioxidant capacity [53,63,64]. In this assay, the contribution of the antioxidant enzymatic system contribution [65,66] is not valued, and this should be taken into consideration.

The analysis of the enzymatic antioxidant system confirmed a dose-dependent increase in the enzymatic activities of SOD, CAT, GPx, and GST in the myotubes after MOLE administration. We have previously demonstrated that MOLE induces the SIRT1-Nrf2 system [29]. SIRT1 acts as a modulator of Nrf2 [67]. Nrf2 is an important transcription factor that potentiates the cellular defense system through its ability to bind and regulate the antioxidant-responsive elements (AREs) [68,69,70]. It is well-known that Nrf2 activation prevents oxidative damage, and its signaling plays a key role in the oxidative-stress-mediated beneficial effects of exercise [71]. When a redox status imbalance occurs, Nrf2 dissociates from its sequestration complex and then translocates to the nucleus. In this way, it can interact with the AREs of antioxidant genes, leading to the transcriptional activation of its target genes such as superoxide dismutase, glutathione peroxidase, glutathione S-transferase, and heme oxygenase-1 [72,73,74]. 

Here we found that MOLE pretreatment is able to maintain the activity levels of all antioxidant enzymes in myotubes exposed to oxidative insult. Our data show that MOLE positively modulates SOD, an enzyme that catalyzes the dismutation of superoxide radicals to generate hydrogen peroxide, which was restored to the level found in the controls. Interestingly, a stronger effect of MOLE was found in the myotubes exposed to H_2_O_2_, with the induction of CAT and GPx, enzymes involved in the neutralization of H_2_O_2_ to H_2_O in peroxisomes, and the cytosol, respectively, with respect to the myotubes challenged with oxidative insult alone. Additionally, the activity of GST, involved in the detoxification of molecules through the formation of S-conjugates with GSH, was found to be significantly increased compared to the myotubes treated with H_2_O_2_ and higher than the control values. 

Our results can be explained by the fact that MOLE is an inducer of cellular antioxidant systems, in addition to acting as a direct scavenger of ROS. Given that MOLE exerts its protective antioxidant effect against H_2_O_2_-induced oxidative stress only in pretreated cells, we speculate that the activation of the Nrf2 pathway is particularly important in enabling cells to respond when subjected to oxidative stress promptly. In fact, our data on muscle cells are in agreement with recent reports on the effects of the different components of *Brassicaceae*, and *Moringa oleifera* in particular, on other cellular models exposed to oxidative stress [75,76,77,78,79,80,81].

Finally, these data allow us to assert that *Moringa oleifera* leaf extract represents a useful nutritional supplement for preventing harmful oxidative stress conditions in skeletal muscles. In doing so, the compounds in MOLE may exert antioxidant activity either directly by scavenging ROS or indirectly by inducing an antioxidant response, thereby ameliorating the cellular redox status. 

## 5. Conclusions

The present study shows that MOLE pretreatment has a beneficial effect on the antioxidant system of skeletal muscle cells, increasing enzymatic capacity and redox status in the stressful condition of an oxidizing environment. 

Our data encourage further studies to better elucidate the action of MOLE on the redox state of different physiological and pathological states in humans in order to generalize the role of MOLE as a supporter of skeletal muscle health.

## Figures and Tables

**Table 1:** Total free thiols and Thioredoxin activity analysis.

	CTRLm	MOLE 1/1000	MOLE 1/100	H_2_O_2_	MOLE 1/1000 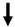 H_2_O_2_	MOLE 1/100 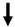 H_2_O_2_
**TFT ^a)^**	**3.69 ± 0.02**	3.94 ± 0.08 *	4.07 ± 0.05 *	3.56 ± 0.03 *	**3.82 ± 0.11 #**	**4.03 ± 0.25 #**
**Total Trx ^b)^**	**20.46 ± 0.75**	22.46 ± 0.28 *	24.31 ± 0.06 *	17.80 ± 0.77 *	**19.44 ± 1.41**	**21.91 ± 0.80 #**
*Active Trx ^b)^*	*3.41 ± 0.17*	4.76 ± 0.71 *	5.59 ± 0.16 *	1.43 ± 0.24 *	2.62 ± 0.03 * #	*3.37 ± 0.20 #*
*Active Trx (%)*	*16.68 ± 0.21*	*21.15 ± 2.29*	22.98 ± 0.61 *	8.04 ± 0.99 *	13.53 ± 0.83 * #	*15.42 ± 1.47 #*

**TFT**, total free thiols; **Trx**, thioredoxin; **Active Trx,** free active form of Trx. C2C12 myotubes were treated with MOLE stock solution dilutions (1/1000 and 1/100 working solution) or vehicle (methanol) in culture media for 24 h. Then, hydrogen peroxide (1 mM) was added to samples pre-treated with vehicle or MOLE for a further hour. After treatments, cells were lysed, and then lysates were used for biochemical analysis. Data presented are the means ± S.D. of three experiments performed in triplicate. a) nmol -SH/g proteins; b) ng Trx/mg proteins. * *p* < 0.05 vs. CTRLm; # *p* < 0.05 vs. H_2_O_2_.

**Table 2:** Enzymatic activity analysis.

	CTRLm	MOLE 1/1000	MOLE 1/100	H_2_O_2_	MOLE 1/1000 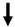 H_2_O_2_	MOLE 1/100 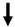 H_2_O_2_
**SOD ^a)^**	**0.35 ± 0.01**	0.38 ± 0.01 *	0.44 ± 0.05 *	0.29 ± 0.02 **	**0.35 ± 0.03 #**	**0.36 ± 0.02 ##**
**CAT ^a)^**	**3.46 ± 0.16**	3.92 ± 0.23 *	4.04 ± 0.09 *	2.97 ± 0.06 **	**3.61 ± 0.13 ##**	**3.81 ± 0.27 ##**
**GPx ^a)^**	**368.49 ± 27.48**	468.20 ± 50.01 *	483.92 ± 31.65 *	312.27 ± 17.17 **	**407.63 ± 16.34 ##**	432.13 ± 22.66 * ##
**GST ^a)^**	**144.07 ± 6.54**	160.26 ± 6.49 *	168.92 ± 3.75 *	123.31 ± 2.61 **	**150.85 ± 5.74 ##**	**± 10.94 ##**

**SOD**, superoxide dismutase; **CAT**, catalase; **GPx**, glutathione peroxidase; **GST**, glutathione transferase. C2C12 myotubes were treated with MOLE stock solution dilutions (1/1000 and 1/100 working solution) or vehicle (methanol) in culture media for 24 h. Then, hydrogen peroxide (1 mM) was added to samples pre-treated with vehicle or MOLE for a further hour. After treatments, the cells were lysed and then cell lysates were used for biochemical analysis. Data presented are the means ± S.D. of three experiments performed in triplicate. a) U/mg proteins. * *p* < 0.05 and ** *p* < 0.01 vs. CTRLm; # *p* < 0.05 and ## *p* < 0.01 vs. H_2_O_2_.

## Data Availability

Data is contained within the article.

## Abbreviations

*Moringa oleifera* leaf extract (MOLE), glucosinolates (GLs), reduced (GSH) and oxidized (GSSG) glutathione, reduced to oxidized glutathione ratio (GSH/GSSG), total antioxidant capacity (TAC), superoxide dismutase (SOD), catalase (CAT), glutathione peroxidase (GPx), glutathione transferase (GST), total free thiols (TFT), thioredoxin (Trx), thiobarbituric acid reactive substances (TBARS), protein carbonyls (PrCar), reactive oxygen species (ROS).
